# Relieving Compression-Induced Local Wear on Non-Volatile Memory Block via Sliding Writes

**DOI:** 10.3390/mi14030568

**Published:** 2023-02-27

**Authors:** Kailun Jin, Yajuan Du, Mingzhe Zhang, Zhenghao Yin, Rachata Ausavarungnirun

**Affiliations:** 1 School of Computer and Artificial Intelligence, Wuhan University of Technology, Wuhan 430070, China; 2 Shenzhen Research Institute, Wuhan University of Technology, Shenzhen 518000, China; 3 Institute of Information Engineering, Chinese Academy of Sciences, Beijing 100190, China; 4 Sirindhorn International Thai–German Graduate School of Engineering, King Mongkut’s University of Technology North Bangkok (KMUTNB), Bangkok 10800, Thailand

**Keywords:** bit flip, memory compression, local wear, non-volatile memories

## Abstract

Due to its non-volatility and large capacity, NVM devices gradually take place at various levels of memories. However, their limited endurance is still a big concern for large-scale data centres. Compression algorithms have been used to save NVM space and enhance the efficiency of those lifetime extension methods. However, their own influence on the NVM lifetime is not clear. In order to fully investigate the impact of compression on NVM, this paper first studies bit flips involved in several typical compression algorithms. It is found that more bit flips would happen in the shrunken area of a memory block. This induces the phenomenon of intra-block wear unevenness, which sacrifices NVM lifetime. We propose a new metric called *local bit flips* to describe this phenomenon. In order to relieve the intra-block wear unevenness caused by compression, this paper proposes a sliding write method named SlidW to distribute the compressed data across the whole memory block. We first divide the memory block into several areas, and then consider five cases about the relationship between new data size and left space. Then, we place the new data according to the case. Comprehensive experimental results show that SlidW can efficiently balance wear and enhance NVM lifetime.

## 1. Introduction

With the rise of big data applications, the amount of data quickly increases, which stimulates the requirements for large memory storage capacity. Traditional memory technologies such as Dynamic Random Access Memory (DRAM) or Static Random Access Memory (SRAM) can not be further expanded due to their manufacturing process. Due to its advantages, such as non-volatility, high storage density and low power consumption, Non-Volatile Memory (NVM), such as Phase Change Memory (PCM) [1,2,3], Spin-transfer-torque Random Access Memory (STT-RAM) [3,4], Ferroelectric Random Access Memory (FeRAM) [3,5] and Resistive Random Access Memory (ReRAM) [3,6], have been widely studied and considered to be an alternative for various levels of the memory hierarchy, e.g., cache, main memory and secondary storage. However, in the existing research, due to the advantage of the large capacity of NVM, large-scale applications such as the nervous system and image processing are usually implemented in the NVM environment. These applications generate large amounts of data, resulting in a large number of bit flips, which speeds up the wear of NVM blocks and jeopardizes the lifetime of NVM devices. NVMs, such as PRAM or STTRAM, are also applied to embedded devices. When applied to embedded systems, the non-volatility of NVM will cause some security problems; the use of encryption algorithms will increase the bit flips and reduce the lifetime. In addition, the endurance of NVM is quite limited with only $10^{8}$ to $10^{9}$ write cycles compared to DRAM with $10^{15}$ writes [3]. Therefore, using NVM as DRAM might result in lifetime issues.

There are many studies on extending the lifetime of NVM, e.g., by keeping wear leveling among memory blocks [7,8,9,10], reducing the number of writes [11,12] or reducing the bit flips [13,14,15,16]. However, these methods often consume extra NVM space for the metadata, e.g., tag information in the most commonly used FNW method that is used to reduce bit flips [15]. Compression algorithms have already been used to save NVM space for existing lifetime extension methods [16,17]. The most popular memory compression algorithms include Frequent Pattern Compression (FPC) [18], Base-Delta-Immediate Compression (BDI) [19,20] and Frequent Value Compression (FVC) [21], which reduce the size of the data by de-duplicating or deleting consecutive “0” s and “1” s. However, the effect of compression algorithms themselves on NVM has not been studied in existing works.

To investigate the effect of compression on NVM lifetime, we conducted a preliminary study with three popularly used compression algorithms. From the study results, it is first observed whether the number of bit flips may increase or decrease after applying compression, which has no direct connection with the compression ratio. As compressed data are often stored in a fixed range of one memory block, only the shrunken area would be affected by bit flips, which would cause block wear locally. To identify and characterize the non-even wearing, we propose a new metric called *local bit flips* to describe the local wear on shrunken areas induced by compression. Furthermore, we observe that all compression algorithms would cause an increased number of *local bit flips*, which would sacrifice the NVM lifetime.

Based on these observations, we propose an intra-block wear-leveling mechanism called SlidW to relieve the local wear effect and enhance the NVM lifetime. The key idea is to distribute the compressed data into different places inside one memory block in a round-robin way. The memory block is first divided into several areas, and the compressed data to be written would be placed into these areas by considering five cases about the relationship between the left space and sizes of old data and new data. For each case, different write data placement methods are applied.

Our experimental results show that SlidW can significantly reduce the *local bit flips*, which leads to a 23.61% reduction in local wear and an overall increase in NVM lifetime. Meanwhile, SlidW can reduce read and write latency by 6.52% and 2.78% and reduce energy consumption by 0.64%.

Our contributions are listed as follows:

- We propose a new metric, *local bit flips*, to describe the effect of compression on local areas of one NVM memory block. From the preliminary study based on this metric, we find that severe local wear is caused by existing compression algorithms, which would sacrifice the NVM lifetime.
- To address the local wear problem, we propose an intra-block wear leveling method called SlidW, which places the compressed data into different areas inside one block. We design the data placement policy under five cases by considering the differences between the size of new data and old data.
- We evaluate our proposed SlidW method using gem5 and NVMain simulators. Experimental results verify that SlidW is able to reduce the local wear effect and extend the NVM lifetime.

The rest of this paper is organized as follows. Section 2 introduces the basics of NVM and existing memory compression algorithms and presents the motivation for our work. Section 3 presents the definition of the proposed new metric for local wear and shows the results of the new metrics after using compression. Section 4 presents the details of the proposed SlidW method. Section 5 describes the platform configurations and analyzes experimental results. Section 6 presents related works to this paper and Section 7 concludes our work.

## 2. Background and Motivation

In this section, we first introduce the basic structure of NVM. Then, we present several commonly used memory compression algorithms. Finally, the preliminary study that motivates our work is illustrated.

### 2.1. Introduction of Phase Change Memory

**Figure 1:**
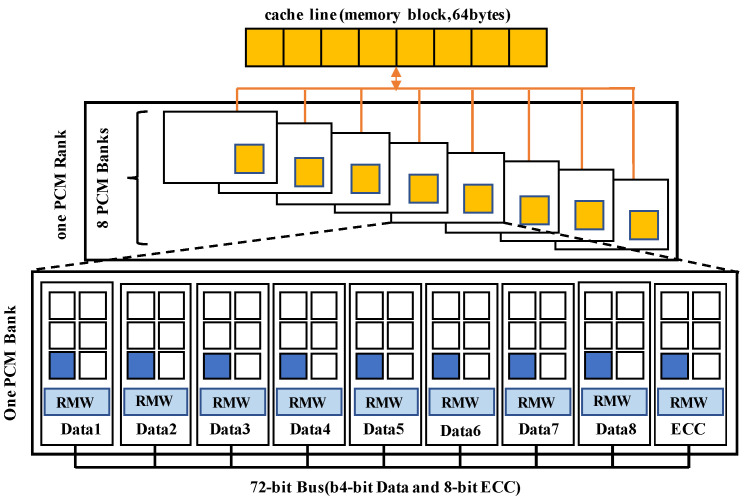
The basic structure of PCM. One bank consists of 8 data blocks and one ECC block.

Among the different types of NVMs, PCM is regarded as a candidate for memory due to its Byte addressabilities such as DRAM, good scalability and low energy consumption. A PCM device has one or multiple channels, each of which is connected to a Dual In-line Memory Module (DIMM) and composed of one or multiple ranks. Figure 1 shows the basic structure of PCM, each rank is composed of eight banks, each of which can deal with memory requests independently. The data in a memory block (cache line) are distributed among eight banks, and each bank provides part of the data [22]. As shown in the bottom of Figure 1, a bank has eight data sub-blocks and one ECC (Error Correcting Code) block [23,24]. The ECC block is used to correct data faults during storage or transfer. When a memory request comes, the blocks provide 8 Bytes of data and 1 Byte of ECC data. These 8 Bytes (blue block in 8 data blocks) make up the small yellow block in the middle of Figure 1. It should be noted that PCM can write cells that are different between old and new data and do not write cells that are the same.

### 2.2. Memory Compression

AS eBay uses two data warehouses at 7.5 petabytes and a 40 PB Hadoop cluster. It is much larger than the capacity of highly dense NVM-based memories (24 × 256 GB). In order to improve storage efficiency and extend memory capacity, compression is widely used in memory systems. The most popularly used memory compression algorithms include FPC, BDI and FVC.

#### 2.2.1. Frequent Pattern Compression

The FPC algorithm divides a memory block into 4-byte words and matches these words with seven general data patterns. These patterns are shown in Table 1.

For instance, the zero run pattern refers to the bits in the word all being zero. The 4-bit sign-extended pattern means that the first 28 bits in the 4-byte word are the same as the 29th bit. For example, the last four bits of the word “0x0000 0007” are “0111”, and the first 28 bits are the same as the 29th bit “0”. These 28 bits are called sign-extended bits.

**Figure 2:**
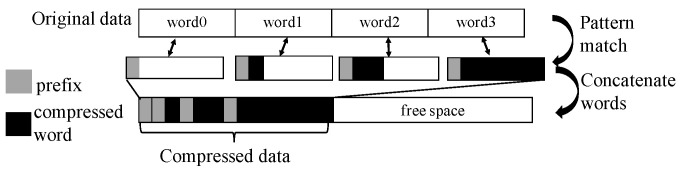
FPC compression process. The light grey area is the prefix. The black area is the compression data. In the process, first the data are divided into words and then matched with the seven patterns; then, the data are compressed with the patterns. Last, the prefix and words are concatenated in turn to form the compressed data.

Figure 2 shows the compression process. Once any of these seven patterns are detected in the word of original data, FPC would first take the prefix in Table 1 as the first three bits to indicate which pattern it matches. Then, FPC would compress the word according to the matched pattern, and put the compressed bits after the prefix to form the compressed word. The word size after compression for each matched pattern is listed in Table 1. Any other patterns that do not satisfy the listed seven patterns are left uncompressed. For instance, if it matches the “zero run” pattern, FPC directly replaces the 4-byte word with the prefix “000”. The compressed size of the original word is 0. For the word “0x0000 0007” that matches the 4-bit sign-extended pattern, the compressed word would be “0010111” in binary, and the compressed size would be 4 bits.

#### 2.2.2. Base-Delta-Immediate Compression

BDI compression has nine patterns of compressing memory blocks (e.g., a cache line often with 64 Bytes), and these patterns can compress data at the same time. These nine patterns are shown in Table 2. #Byte refers to the number of Byte.

The zero pattern will judge whether the data in the memory block are all zero. If so, then the data will be compressed into 1-byte zero data. The repeated values pattern will divide memory data into 8-byte words and judge whether all 8-byte words are the same. If so, the data will be compressed into the first 8-byte word. The Base8-Delta1 pattern will divide memory data into 8-byte words, choose the first word that minus zero is larger than the 1-byte data as the base, and calculate the delta between the divided words and the base or zero. If all the delta can be represented in 1 byte, then the data will be compressed into data that puts all 1-byte deltas together after the base. For example, the base is 0x1234, and one of the divided words is 0x1235, the delta is 0x01 and it can be represented in 1 byte, but the other divided word is 0x3234, the delta is 0x2000, and it cannot be represented in 1 byte, so the memory block cannot be compressed in this pattern. The Base8-Delta2 pattern and Base8-Delta4 compressed pattern are the same as Base8-Delta1, but the delta should be represented in 2 bytes and 4 bytes, respectively. The Base4-Delta*Y* and Base2-Delta*Y* are to divide memory data into 4-byte words and 2-byte words, respectively, and the Y denoting the delta should be represented in Y bytes; the compressed data consist of the base and Y-byte delta.

**Figure 3:**
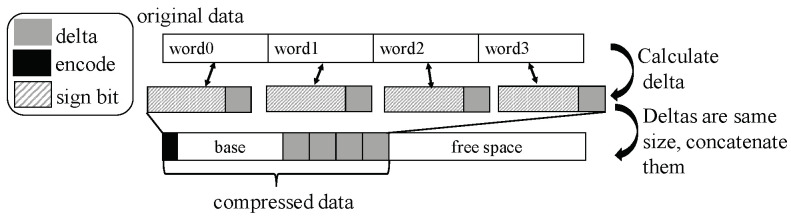
BDI compression process. The black area is the encode, the grey area is delta and the diagonal stripes are the sign bits. In the process, first, data are matched with these patterns, then data are compressed with these patterns; last, the prefix and words are concatenated in turn to form the compressed data.

In the compression process, BDI matches the above-mentioned patterns simultaneously and chooses the matched pattern with the smallest compressed size. Then, the encoded pattern is placed at the beginning of the compressed data, as shown in Figure 3. The last column of Table 2 shows the size after compression by each pattern for the 64-byte block size.

#### 2.2.3. Frequent Value Compression

**Figure 4:**
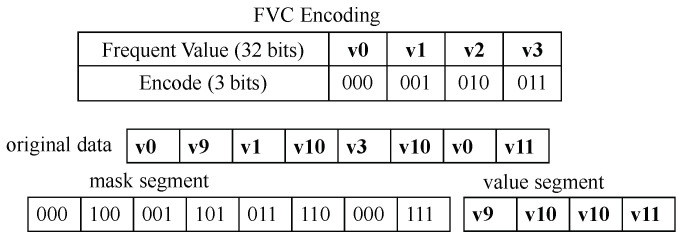
An example of FVC compression. The FVC encoding table at the top of the figure gives an example of frequent values and their encodes. The mask segment stores the encode or site of data, and the value segment stores the infrequently used value.

Just like the FPC algorithms, the FVC compression algorithm also divides a memory block into 4-byte words and performs the compression within two stages. First, FVC samples the most frequently used words and encodes them into a 3-bit encode. The first bit “0” of the encode indicates that it is a frequent value, and the last two bits represent the position of the frequent value in the FVC encoding table. Figure 4 shows the case with the four most frequently used words. Second, FVC compares each word with frequently used words and uses two segments of the mask and value segments to differentiate frequent and infrequent words. If the word belongs to frequent values, FVC does not store the word in the value segment but puts its encoded pattern to the mask. Otherwise, the data are stored in the value segment and the value segment position is put into the mask segment. In the example of Figure 4, as the first word v0 belongs to the frequent word, it would not be stored in the value segment. The first position in the mask segment would store the encode of v0. The second word v9 is not frequent data, so it is placed in the value segment, and its position (the first position “00”) in the value segment is stored in the mask segment. Note that the bit “1” in front of “00” is used to indicate infrequent words.

### 2.3. The Preliminary Study

As shown in Section 2.2, memory compression algorithms destroy the original data organization, which may change the number of bit flips caused by data writing. In order to investigate the effect of compression on bit flips of NVM, this section performs a preliminary study on the three above-mentioned compression algorithms. We conduct experiments on gem5 and NVMain and run nine traditional benchmarks. The detailed experimental configuration is listed in Section 5.

**Figure 5:**
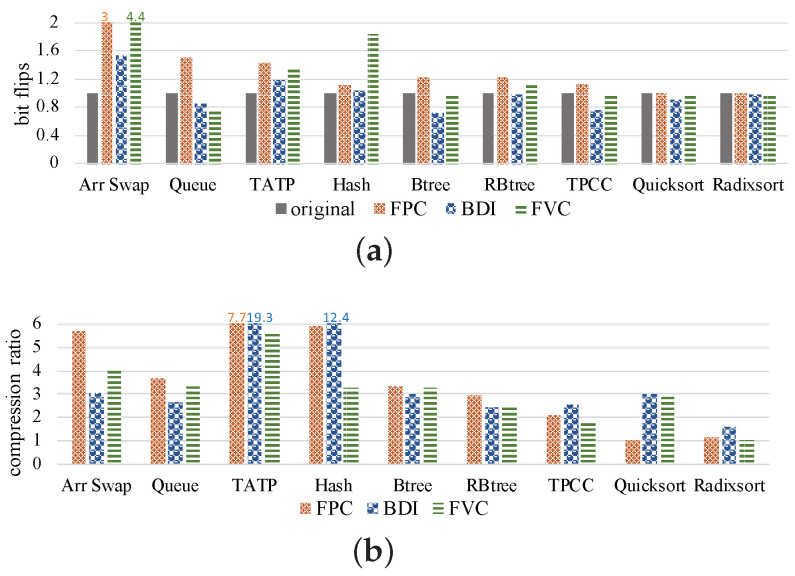
Results of the preliminary study under three compression algorithms. (**a**) The result of bit flips normalized to original data. (**b**) The result of compression ratio.

To study the relationship between bit flips and compression ratio, we collect the number of bit flips that happen when the original value in the cell is not equal to the value to be written, and the compression ratio is equal to the original data size divided by the compressed data size. As shown in Figure 5, we can obtain two observations. First, compression algorithms increase bit flips in some benchmarks, especially for the benchmarks *Arr Swap, TATP* and *Hash*. Compared with the original data, FPC, BDI and FVC increase the number of bit flips by 59.3%, 4.84% and 72.92% on average, respectively. This means that although compression reduces data size, the bit flips would not be reduced but may increase. During the benchmarks with high compression ratios, e.g., *TATP*, the bit flip number surprisingly increased significantly. Second, when different compression algorithms are used for the same benchmark, there is no relationship between compression ratio and bit flips. For example, in *TPCC*, the BDI compression algorithm provides the highest compression ratio but its bit flip number is the lowest, while the FVC compression algorithm is the worst in the compression ratio, but its bit flip number is not the highest.

The above results and analysis show that compression may lead to an increase or decrease in bit flips. Moreover, the number of bit flips can only represent the average wear of the memory block. However, the compressed data are usually stored in a shrunken area in the memory block (often focused in the first half of the block). Therefore, compression would induce uneven wear inside a memory block.

Because of using compression, the bitflip will be reduced due to the reduction in size, but the bitflip will be concentrated in a small area of the block, resulting in an increase in the flip of local cells. If there is failure in one cell in the block, the entire block also fails. Traditional bitflip shows the total bit flips in one block, but we can not know where the bit flips happen. Same-bit flips have different effects in small and big areas, so it is not applicable. In order to solve this problem, we first propose a new metric named *local bit flips* to evaluate the effect of compression in the shrunken area. Details are presented in the next section.

## 3. The Local Bit Flips

In this section, we first present the definition of the proposed *local bit flips* to better evaluate the local wear caused by compression. Then, we show the experimental results of this metric in the preliminary study and discuss the motivation for proposing SlidW.

### 3.1. The Definition of Local Bit Flips

**Figure 6:**
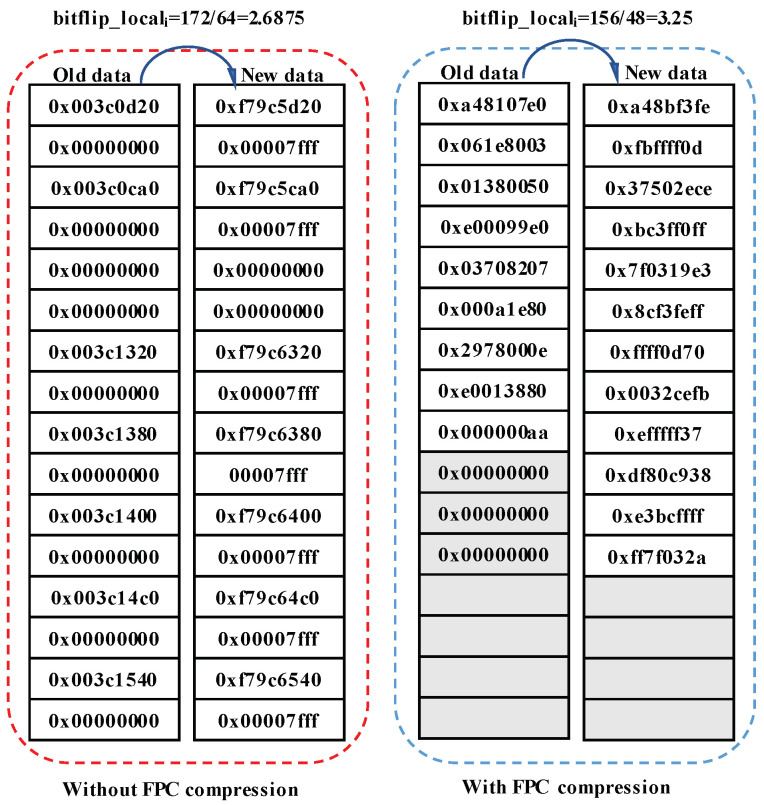
An example of an increase in *local bit flips* occurred in one NVM write taken from the *TATP* benchmark. The *local bit flips* for this write are computed with and without compression.

Before presenting the definition of *local bit flips*, we first discuss what happens in the compression with an example shown in Figure 6. The data are collected from the benchmark *TATP* and the compression algorithm is FPC. In the original block without FPC compression, when old data are replaced by new data, some bits would be flipped. The bit flips happen across the whole memory block. When FPC compression is applied, the memory areas taken by old data and new data are both shrunken. In this situation, the bit flips only happen in the shrunken area. Thus, it should consider the size of area in which bit flips happen.

(1) $$bitflip\_local_{all}=\sum_{i=1}^{n}bitflip\_local_{i}=\sum_{i=1}^{n}\frac{bitflip_{i}}{datasize_{i}}$$

(2) $$lifetime=\frac{Endurance}{bitflip\_local_{all}}$$

We define the *local bit flips* as the number of bit flips per unit area. The equation for calculating the *local bit flips* represented as $bitflip\_local_{all}$ is in Equation (1), wherein the *n* and *i* represent the total number and the times to write the sequence of writes, respectively. $bitflip\_local_{i}$ refers to the number of *local bit flips* in the $i^{th}$ write. $bitflip_{i}$ and $datasize_{i}$ show the number of bit flips and the size of area in which these bit flips happen during the $i^{th}$ write. In the example of Figure 6, the *local bit flips* are calculated according to Equation (1). We find that the *local bit flips* increased after using FPC compression, which means that compression induces worse local wear on NVM blocks. In order to further describe the effect of *local bit flips* on the lifetime of NVM, the lifetime is defined as Equation (2). Endurance is the maximum number of writes that can be made in a cell.

### 3.2. Results of Local Bit Flips

**Figure 7:**
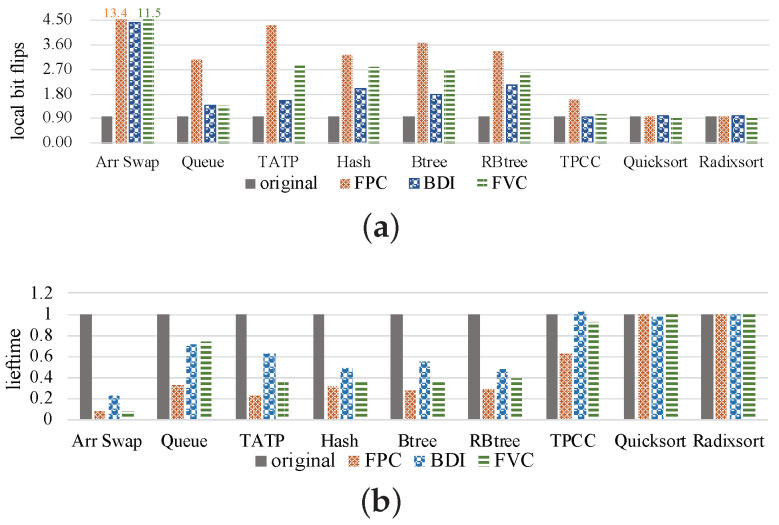
Results of the preliminary study using three compression methods. (**a**) Local bit flips, (**b**) NVM lifetime.

In order to show the effect of local wear, we collected more data about the number of *local bit flips* and NVM lifetime in the preliminary study, and the results are shown in Figure 7. In this figure, we first observe that most benchmarks with three compression algorithms would increase the number of *local bit flips*. This is because when compression is used, the negative impact of the reduction in data size is severely exaggerated. Especially for benchmarks in which compression increases bit flips, this negative effect would be further worsened. Meanwhile, the increase in local wear will reduce the lifetime of NVM. As shown in Figure 7b, when the *local bit flips* increase, the lifetime decreases. Second, we observe that for some benchmarks, the *local bit flips* are not obviously increased by compression, e.g., in Quicksort and Radixsort. For the benchmark TPCC, BDI reduces the number of *local bit flips*. This may be because the positive effect of data bit flips reduction is greater than the negative effect of size reduction.

Third, the FPC algorithm increases the bit flips in all benchmarks, as shown in Figure 5a, and also induces the largest local wear effects among the three compression algorithms.

The above experimental results show that the area size reduction caused by compression induces severe wear in the shrunken area. Thus, the wear inside the whole memory block is not even, which would sacrifice the NVM lifetime. In order to avoid this uneven local wear effect, we propose an intra-block wear leveling method to disperse the local wear into the whole memory block. Details will be presented in the next section.

## 4. The Sliding Write Method

This section details the proposed SlidW. First, its architectural overview and the read/write streams are introduced. Then, the SlidW components are illustrated. At last, we discuss the overhead involved in SlidW.

### 4.1. Overview

**Figure 8:**
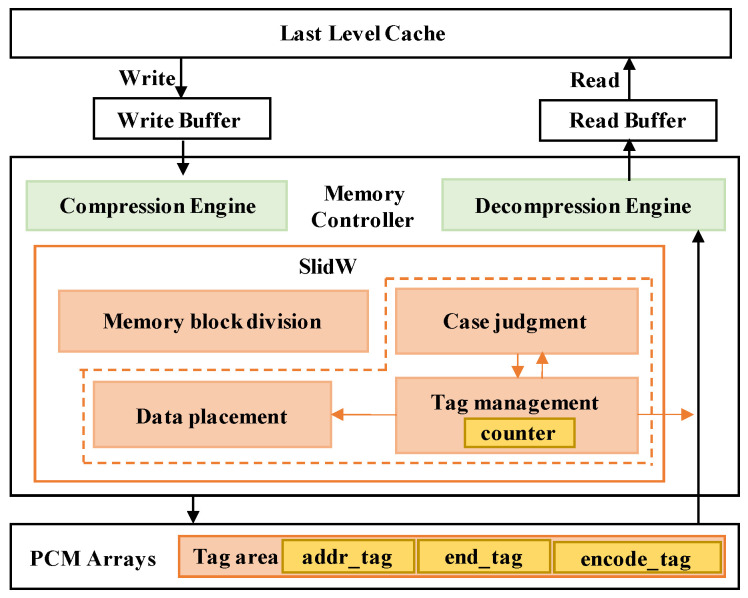
The architectural overview of SlidW.

In order to solve the local wear problem of NVM caused by compression, SlidW deals writes with a sliding window. The basic design is to place compressed data into different areas of the memory in a round-robin way. In detail, SlidW contains four modules of memory block division, case judgment, tag management and data placement, as shown in Figure 8. In the first module, the memory block is divided into several areas. The compressed data size is not fixed, it may be larger or smaller than the area. SlidW then considers five cases about the size relationship among area size, old data and new data in the case judgment module. SlidW uses several tags to indicate the written case and manage them in the tag management module. Once the case is determined, SlidW would decide how to set the tag. Finally, the data placement module would decide the place to write data according to the tag information and send the data together with tag information to the NVM Array. Note that the tag information is stored in the ECC area or extra memory blocks of PCM arrays.

During the writing process, data would be first compressed in the compression engine. Then, compressed data would be dealt with in the SlidW module to decide how to place the data and process the data accordingly. At last, the data are sent to the PCM arrays to finish the write operation. During the reading process, data would be first read out together with the tag. According to the assistance of the tag management module in SlidW, address and status information would be identified and the data would be parsed and organized, then sent to the decompression engine to finish the reading process.

### 4.2. Memory Block Division

In order to fully balance the wear caused by compression, the best way is to allow data to be written from the place in a block without overlapping, that is, to place the newly written data exactly at the end of the old data. However, this requires storing a lot of metadata to identify valid parts of the data, and processing the data is also more complicated. In order to reduce the storage of metadata, we divide the data into four areas, so that it can be stored from several positions, thereby simplifying the metadata and saving space. The memory block division module is responsible for logically dividing the physical memory blocks into areas of the same size. The number of areas depends on the granularity of writes and can be adjusted according to the requirement of writing sizes. For example, if the granularity is 4, the physical memory block is divided into four areas. Assume that the address range of the memory block to be written is from “0x00000000” to “0x00000040” (64 Bytes), and the size of each area is 16 Bytes. The logical number of the four areas is denoted as “00”, “01”, “10” and “11”, respectively.

### 4.3. Case Judgment

The case judgment module judges which case to use and determines the data placement strategy. As we find in the experiment, there are many zero blocks that will be written to memory, such as initialization of an array, which will cause additional zero writes, so we consider this situation separately. To fully balance the data into blocks, we try not to overlap the data. Therefore, whether the new data can be written to the next part of the old data will be considered. It is a situation in that data can be written to the next part of the old data. If data cannot be written to the next part of the old data, and it is written from the beginning of the block again, this results in the last area rarely being written. If it is written from the back, the front space will be rarely written. According to the size of the remaining space and the size of the newly written data, SlidW will consider two situations. In the above situation, the writing of the different areas is relatively uniform. However, considering some extreme cases, such as a large gap between old and new data compression rates, the newly written data cannot be wear-balanced with the old written data. In this situation, the one with a small compression ratio can still be written normally, and the last space in the block is rarely written. However, the one with a large compression ratio can be written to the last space to better balance. In summary, we divide the write into five cases.

The compressed size of data to write is first provided to the case judgment module by the compression engine. The case judgment then calculates the left space according to the tag information of the old data and decides write cases. The calculation of the left space is to subtract the end position of data from the granularity and then multiply the result by the area size. The area size is calculated by memory block size divided by granularity. A shift operation can replace the process of multiplying and division. As mentioned above, SlidW considers five cases according to the information of new data size, left space and the counter that tracks the consecutive write number that writes from the first area of the memory block.

In the first case, the new data size is less than a threshold denoted as $T_{size}$ and less than the left space. We can just put data after old data, so this will not cause overwriting. Note that $T_{size}$ is computed as $T_{size}=blocksize*\left(granularity-1\right)/granularity$. In the second case, the new data size is greater than the left space and less than threshold $T_{size}$, but the left space is larger than zero. It can be considered that compared with rewriting from the 00 position (the size of the space that needs to be covered is the size of the new data), writing to the writable position is more space saving (the coverage area is the new data size minus left space). Therefore, we separate this case out. In the third case, the new data are all zero. We can skip this write to reduce the number of times that the block is written. In the fourth case, the new data size is smaller than threshold $T_{size}$, and the counter has reached threshold $T_{counter}$. Note that data would be written from the end of block in both the second and fourth cases, i.e., in reverse order. This is because most writes start from the beginning of each data area, which would make the wear worse in the first part of data areas. Using the reverse write can avoid this situation. In the fifth case, the new data set is larger than $T_{size}$. Then, we just write from the beginning.

**Figure 9:**
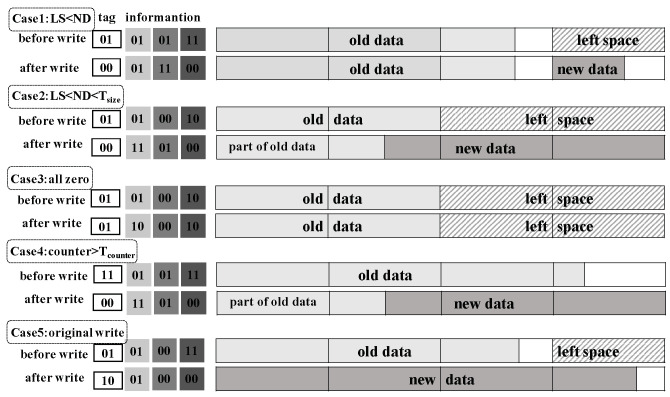
An example of 5 write cases under the granularity of 4 areas. Different write policies would be taken by considering the cases. The first row in each case shows the state of memory block before writing, while the second row shows the block state after writing the compressed data. Light grey square is encode_tag, medium grey square is addr_tag, dark grey square is end_tag, white square is counter. Case1: left space size is 16 Bytes, new data size is 10 Bytes; Case2: left space size is 32 Bytes, new data size is 40 Bytes; Case3: write zero lines; Case4: counter equals 3, the new data size is 40 Bytes; Case5: the new data size is 60 Bytes.

Figure 9 shows an example of five cases in the situation where the memory block is divided into four areas, and the two thresholds $T_{size}$ and $T_{counter}$ are 48 and 3, respectively. LS means left space. ND means new data size.

The process of the case judgement algorithm is shown in Algorithm 1, in which NSize is obtained by dividing the new data size by area size. For example, when the data size of the new data written is 35, NSize= ⌈35/16⌉ = 3. When data and some supplementary information such as data size and tag information are sent to the case judgment part, it would first judge whether the data are all zero. In FPC and BDI, we can judge whether the data of the memory block is all zeros according to the data size because the data size of all zeros is fixed to a unique value. When the FVC algorithm is used for the other algorithm, that data size cannot be used to judge whether it is all zeros; thus, we need to judge it in the compression engine and pass this message to the case judgment module. For all zero data, case judgment will determine that this is Case3. Otherwise, case judgment will judge whether the data size is less than or equal to the set threshold $T_{size}$. If so, case judgment decides the written case according to the size relationship among the left space, new data size and counter value. If the left space is larger than or equal to the new data size, case judgment will determine that this is Case1. Otherwise, if the left space is larger than zero, case judgment will determine that this is Case2. If still not, it will judge whether the value of the counter is greater than the threshold $T_{counter}$, if so, case judgment will determine that this is Case4. If the data size is smaller than $T_{size}$, case judgment will determine that this is Case5. The decided case and the data size will be passed to the tag management module.


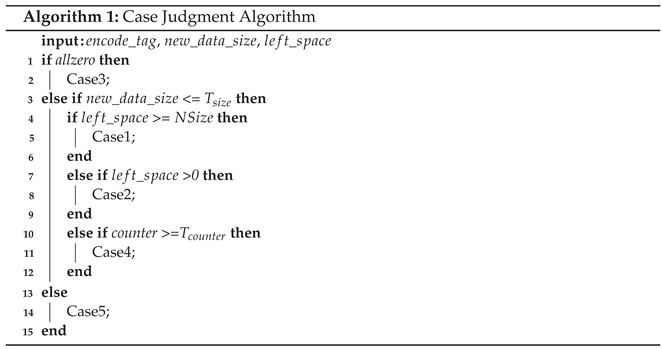


### 4.4. Tag Management

SlidW uses three tags and a counter to indicate write cases and assist reads and writes. They are the encoding tag to indicate the encoding information, denoted as encode_tag; the address tag to indicate the starting position of the data storage, denoted as addr_tag; the end tag to indicate the end position of the data storage, denoted as end_tag; and the counter mentioned above. The counter always takes 2 bits and is set according to the written cases. Encode_tag also always takes 2 bits. The number of bits taken by addr_tag and end_tag depends on the granularity of SlidW. For the situation with four areas, addr_tag and end_tag take 2 bits for each.

Once the case is decided in the case judgment module, the tag management module would set these tags and counters according to Table 3 and Algorithm 2. The modified tag information will be passed to the Data placement module.


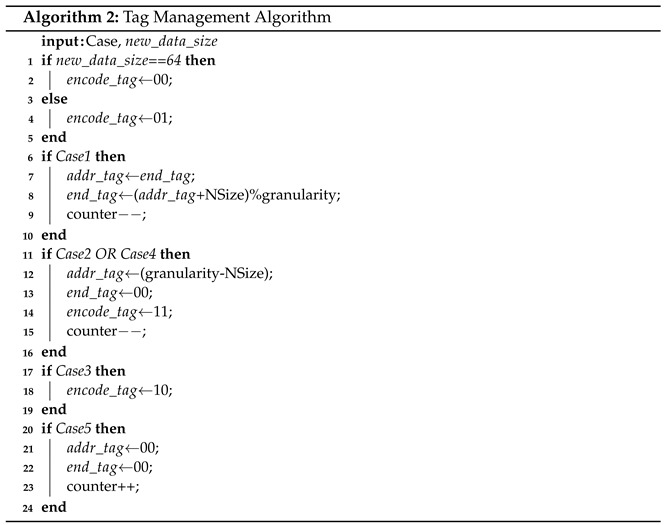


### 4.5. Data Placement

The data placement module is used to decide where to put the data according to the addr_tag calculated by the tag management module and encode_tag. When the encode_tag is set to 11, then the data should be written in reverse from the end of the memory block; that is, the first byte of the data is written into the last byte of the memory block, and then the data are sequentially written to the front bytes of the memory block until the data are written done, and the first few bytes of memory block do not need to be written. When the encode_tag is 10, we just skip this write. When encode_tag is 00 or 01, data placement needs to be performed according to $addr_{t}ag$. For the situation with four areas in the memory block, addr_tag has four states. For the state “00”, new data would be written from the beginning of the memory block. For the other three states, SlidW would rotate the written data to the corresponding area of the memory block. When the writing position is not between the position indicated by addr_tag and the position indicated by end_tag, the cell writing will be skipped. Once the data position is processed, it will be sent to the NVM array together with tag information.

### 4.6. Overhead Analysis

In SlidW, tag information would take extra storage space. We assume that each NVM block is 64 Bytes and equipped with 8 Byte ECC blocks. We take the case with four areas in the memory block as an example. The three tags take 6 bits. They would be first stored in the ECC area when the space used for ECC is less than 58 bits, which means the ECC space is enough to store the tag information. There is no extra data storage space. In the situation that the ECC size is larger than 58 bits (using BCH code to correct 6 errors, it will take 61 bits [24]), tags of SlidW would be stored in extra data blocks, and the overhead is 3 bits per memory block. This overhead is still negligible. After the data are compressed, the required ECC bits will be reduced, so we can have more space to place the tag bits. Tag can also perform wear leveling, such as changing the position at regular intervals, so as to reduce the wear and tear of the ECC. Besides, we need a 2-bit counter in the tag management module, it is negligible.

From the view of algorithm complexity, SlidW involves the data size comparison, write case judgment in data writes and tag management in both data writes and reads. The case judgment module needs the tag as the information to judge the case when data are written. This part of the information is read during compression, so it does not take extra time. As most information can be directly obtained by fast access and computed with O(1) complexity, the effect of SlidW on reading and writing can be ignored.

## 5. Evaluation

In this section, we first present the experimental setup. Then, the results of *local bit flips* and lifetime are illustrated. The granularity sensitivity study and compression algorithm sensitivity study are illustrated. Finally, the results of read/write latency and energy are presented.

### 5.1. Experimental Setup

To verify the effectiveness of SlidW method, we perform comprehensive experiments in the platform built by gem5 [25] and NVMain [26]. The gem5 simulator is a modular platform for computer system architecture research, encompassing system-level architecture as well as processor micro-architecture. NVMain is a cycle-accurate main memory simulator that emulates emerging non-volatile memories at the architectural level. Our experiments use gem5 to simulate the CPU and two-level cache (L1 and L2 cache) structure and use NVMain to simulate PCM as the main memory. The cache line size is set to 64 Bytes. The detailed configuration is shown in Table 4, in which lat. is short for latency. Nine traditional benchmarks [27,28] were used in the experiment and their specific configurations are shown in Table 5, in which *TATP* [29] and *TPCC* [30] are commonly used real-world benchmarks. # in the Table 5 indicates that the number of operations. The parameters of granularity, data size threshold $T_{size}$ and counter threshold $T_{counter}$ involved in SlidW were set to 4, 48 and 3, respectively.

#### 5.1.1. Compared Methods

We first verify the effectiveness of SlidW combined with the FPC algorithm and compare the FPC+SlidW method with three existing methods of FPC, FPC+FNW and FPC+Space. Details are illustrated as follows:

- FPC is the baseline method to directly use the FPC algorithm in NVM. The FPC algorithm is a frequently used compression algorithm. The specific compression process is described in Section 2.2.1.
- Flip-N-Write (FNW) [15] is a method to reduce bit flips by selectively flipping data according to the bit flip number between old data and new data. In detail, it first divides the data in the block into several same-size segments. In this experiment, data are divided into eight segments because n segments require n additional flag bits and using compression will reduce the data size by at least 8 bits. The eight flag bits generated by setting eight segments can be stored in the memory block without taking up additional storage space. The size can be changed according to the requirements. Then, it needs to count the number of bit flips in each segment. If the number of bit flips is greater than half the size of the segment (in bits), it flips the entire segment and sets the corresponding flag bit indicating that the segment is flipped, so that the number of flips per segment is less than half the size. If the number of bit flips is less than half the size of the segment, no flips are performed and the corresponding flag bits are reset. FPC+FNW is to use the FPC algorithm in FNW. In order to facilitate data reading, we place the flag bits in the last byte of the memory block. FPC+FNW is to use FPC algorithm in FNW.
- Space [7] is a method to implement intra-block wear leveling by moving data into different segments. It needs to divide a block into four segments of the same size. Write from the first block for the first time, and then write from the next segment of the last written segment each time. If the data can not write starting from the next segment, it loops forward to the position where it can be written. It will also be skipped if zero lines are written. This algorithm does not consider the size of the old data, resulting in a large amount of data overlap. The writing of the second half and the writing of the first half of the four segments will also be unbalanced. Using the loop algorithm to go forward in turn until finding a location where data can be written, will also increase the time complexity, although, at most, three comparisons. FPC+Space is to use FPC algorithm in the Space method.

#### 5.1.2. Calculation of Local Bit Flips

According to Equation (1) in Section 3.1, *local bit flips* equal the total *local bit flips* involved in each writing. As SlidW distributes the local wear across the whole memory block by dividing the block into areas and data may not be written at the beginning of the block, the definition of *local bit flips* in Equation (1) should be changed to reflect the available data size in SlidW. For example, the new write may be written in the second block area in SlidW. In order to calculate the local wear more accurately, the *local bit flips* should be combined together with the last write. Considering the multiple cases in SlidW, we update the calculation of total *local bit flips* in Equation (3). In Equation (3), *i* indicates several writes that happen in the same memory block but in different areas. *i* would only increase when the memory block is changed or the memory block is written before or at the same position as the last written, and not in reverse. $lbf_{total}$ is the total *local bit flips* and $lbf_{i}$ is the $i^{th}$ *local bit flips*.

(3) $$lbf_{total}=\sum_{i=1}^{n}lbf_{i}$$

The *local bit flips* for each *i* are calculated in Equation (4) by considering different write cases in SlidW. In the equation, as one $i^{th}$ time, there may be old writes and new writes. $bf_{i}^{new}$ and $bf_{i}^{old}$ are used to represent their bit flip number, respectively. $size_{i}^{new}$ and $size_{i}^{old}$ indicate the data size of new write and old writes, respectively. Note that there may be coverage between old data and new data, $csize_{i}$ is used to represent the size of this coverage area in the $i^{th}$ *local bit flips*.

(4) $$lbf_{i}=\left\{\begin{array}{c} \frac{bf_{i}^{new}}{size_{i}^{new}}\;\;addr\_tag_{new}<=addr\_tag_{old}\&\&not\;reverse\\ \frac{bf_{i}^{new}+bf_{i}^{old}}{size_{i}^{new}+size_{i}^{old}-csize_{i}}-\frac{bf_{i}^{old}}{size_{i}^{old}}\;else \end{array}\right.$$

In addition, we also evaluate the effectiveness of our proposed SlidW method, using an existing metric IntraV [31] that calculates the bit flip variance of cells among a memory block. As IntraV only evaluates the average bit flips difference between different cells, but can not reflect the effect of overall bit flips like our proposed *local bit flips* metric.

### 5.2. Experimental Results and Analysis

#### 5.2.1. Local Bit Flips

**Figure 10:**
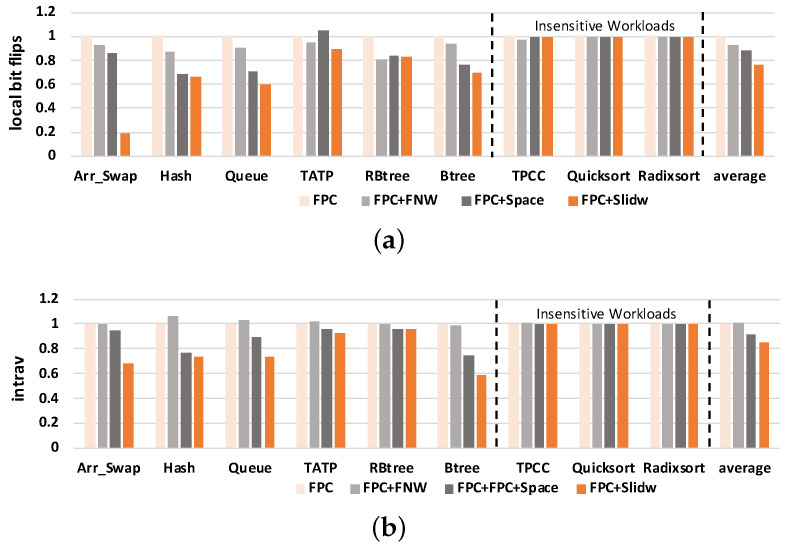
Comparison of NVM local bit flips and IntraV under four different methods. Results are normalized to FPC. (**a**) Results of local bit flips; (**b**) Results of IntraV.

The results of *local bit flips* are shown in Figure 10a. The *y*-axis denotes the *local bit flips*, and all results are normalized to FPC. The figure shows that all three improved methods reduce the *local bit flips* compared with the baseline FPC method. For FPC+FNW, the *local bit flips* are reduced along with the reduction of bit flip number. Both Space and SlidW decrease the *local bit flips* by distributing the local wear into the whole memory block. On average, SlidW can reduce the *local bit flips* by 23.61% in FPC, by 17.98% in FPC+FNW and 13.09% in FPC+Space. Compared with FPC+FNW, the effect of SlidW on relieving the local wear is more obvious. This is because it can put data into more space. This means that the benefit is quite limited to reducing *local bit flips* by only reducing bit flips.

Compared with the FPC+Space method, FPC+SlidW performs better. This is because SlidW fully considers the five cases about the relationships among old data size, left space and new data size. In the case that the size of the old data is greater than the size of an area, FPC+Space would directly place the new data next to the starting position of the old data. FPC+SlidW would deal with this case by selectively putting the new data into the position where the old data ends according to the size of the old data and new data, and it can also put data at the end to further reduce coverage size. Thus, more address changes are induced and a smaller coverage size is created. The lower the coverage size is, the better FPC+SlidW behaves. Table 6 lists the address change times and coverage size of the two methods. FPC+SlidW would induce less coverage in all benchmarks and more address change times in most benchmarks.

This means that FPC+SlidW can make data more evenly distributed in the memory block and its advantage on wear leveling can be fully exploited. The experimental results of IntraV are shown in Figure 10b. Compared with FPC, our method reduces the value of IntraV by 15.23% on average. This also verifies the effectiveness of our proposed SlidW method. In summary, the effectiveness of FPC+SlidW is affected by both address change times and coverage size.

#### 5.2.2. NVM Lifetime

**Figure 11:**
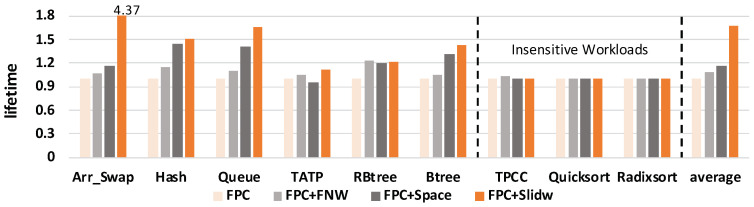
Comparison of NVM lifetime under four different methods. Results are normalized to FPC.

The results of NVM lifetime are shown in Figure 11. The figure shows that all three methods improve NVM lifetime compared with the baseline FPC method. On average, FPC+SlidW can improve NVM lifetime by 67.78% compared with FPC, by 55.63% compared with FPC+FNW and by 44.09% compared with FPC+space, respectively. According to Equation (2), lifetime is inversely proportional to *local bit flips* and proportional to endurance. As endurance is fixed, the lifetime results mainly relate to *local bit flips*. These results verify that our proposed method can effectively improve NVM lifetime.

#### 5.2.3. Sensitivity Study on Block Division Granularity

**Figure 12:**
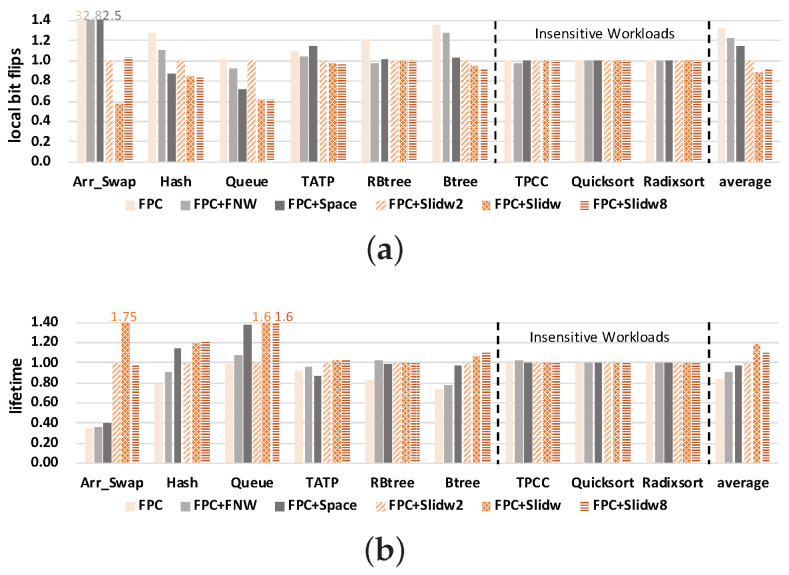
Sensitivity study results of NVM lifetime on block division granularity. FPC+SlidW with three granularities is studied and results are normalized to FPC+SlidW8. (**a**) Results of local bit flips. (**b**) Results of NVM lifetime.

In the primary method, we set a memory block as divided into four areas, i.e., the granularity is 4. This section studies the sensitivity of FPC+SlidW on the other granularity settings of 2 and 8. Figure 12 shows the results of the sensitivity study on the granularity partition.

From the results in Figure 12, it can be first seen that all FPC+SlidW methods with three granularities can reduce the number of *local bit flips* and improve NVM lifetime compared with the other three methods. This verifies that the effectiveness of FPC+SlidW on relieving local wear effect is not significantly affected by the number of block division areas. Then, it can be observed that the results with different granularity show differences in some benchmarks, especially in *Arr Swap* and *Queue*. For most of these benchmarks, the FPC+SlidW method with a larger granularity shows better performance. For example, on average, FPC+SlidW4 reduces 11.69% of the *local bit flips* in FPC+SlidW2. This is because the FPC+SlidW method with a larger granularity generates more address changes. Thus, compressed data can be fully distributed across the whole block and local wear effect can be better relieved. It can also be found from Figure 12 that SlidW with a smaller granularity may behave better for special benchmarks. For instance, FPC+SlidW4 shows a 44.16% reduction of *local bit flips* than FPC+SlidW8 in *Arr Swap*. This is because the FPC+SlidW method with a smaller granularity generates a smaller coverage size.

Finally, different benchmarks behave differently based on different granularities. This is because different benchmarks have different compression ratios, and the memory block size after compression varies with the benchmark. A larger compression ratio suits a smaller granularity. Because a smaller granularity has less area, it can store data from more positions. A larger compression ratio makes data small, so they can take advantage of these positions. So one physical memory block can store more compressed data, but a smaller compression ratio means the data size after compression is big. When using a smaller granularity, SlidW, it will cause a greater coverage size. Besides, when compression ratio is small, the extra start position is not used, and the tag bit will be wasted.

According to the above results and analysis, it can be concluded that FPC+SlidW can effectively improve NVM lifetime and the effectiveness is slightly affected by the block division granularity.

#### 5.2.4. Sensitivity Study on Compression Algorithms

**Figure 13:**
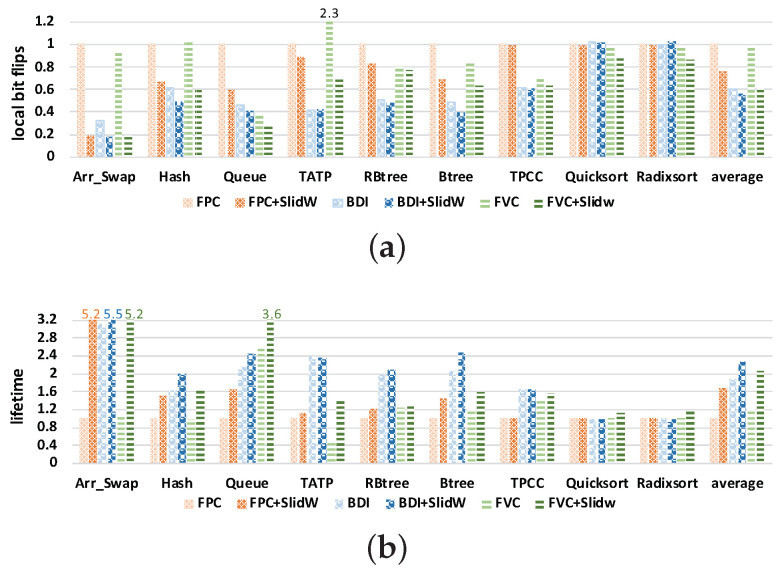
Study results of SlidW on compression algorithms. These results are normalized to FPC. (**a**) Results of local bit flips. (**b**) Results of NVM lifetime.

In the primary method, we show the effectiveness of SlidW combined with the FPC compression algorithm. This section studies the performance of SlidW combined with the other two algorithms of BDI and FVC. The study results are shown in Figure 13; these results are normalized to FPC. From the figure, it can be first seen that SlidW can reduce the number of *local bit flips* and improve NVM lifetime when combined with different compression algorithms. On average, SlidW can reduce *local bit flips* of FPC by 23.61%, that of BDI by 7.04% and that of FVC by 37.70%, respectively. We find that different compression algorithms behave differently when using SlidW because the different compression algorithm has a different compression ratio and number of *local bit flips* compared to without using compression. When the compression ratio is large, it may increase *local bit flips*, and SlidW will be more efficient. These results verify that SlidW can effectively relieve local wear effect caused by compression algorithms and improve NVM lifetime.

#### 5.2.5. Sensitivity Study on Block Size and Cache Level

**Figure 14:**
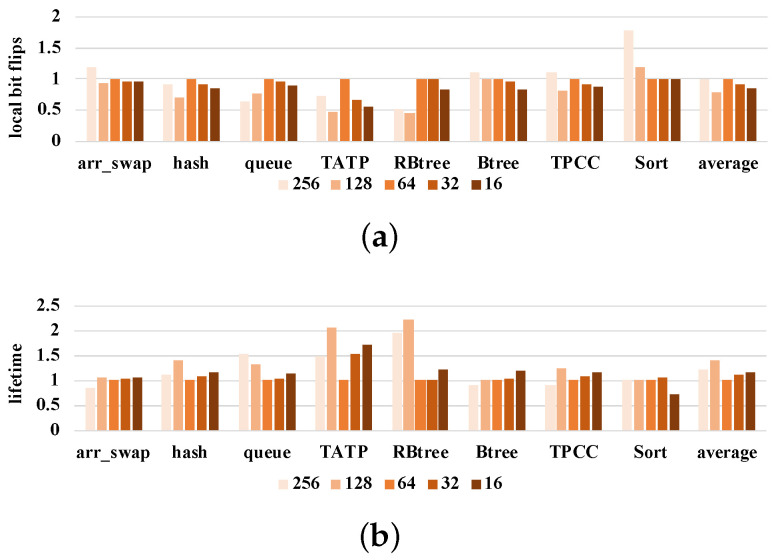
Study results of SlidW on different block sizes. These results are normalized to 64 bytes. (**a**) Results of local bit flips. (**b**) Results of NVM lifetime.

In order to study the impact of different cache systems on our method, we added a third-level cache, the capacity of l3cache was set to 8 MB, and the l2cache was changed to private cache and changed to 256 KB. In order to increase the memory pressure, we increased the number of reads and writes of the benchmark and combined the two sorting algorithms into a Sort benchmark. The results are similar to those with a 2-level cache. SlidW is 23.24% better than FPC, 18.17% better than FNW and 15.32% better than space. At the same time, the lifetime increased by 73.16%, 62.16% and 53.60% compared with FPC, FNW and space, respectively. On this three-level cache architecture, we adjusted the block size. As shown in Figure 14, we can see that SlidW works better than the 64-byte block in some block sizes. SlidW works best on 128-byte blocks and worst on 32-byte blocks.

#### 5.2.6. Results of Read and Write Latency

**Figure 15:**
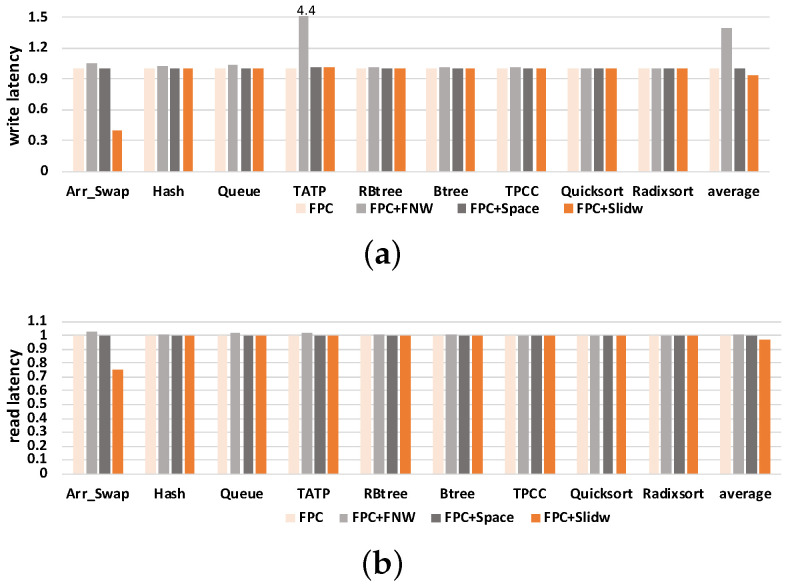
Results of the read/write latency under four compared methods. (**a**) Write latency; (**b**) read latency.

This section checks the effect of SlidW on read/write latency of NVM. Figure 15 shows the results of read and write latency for different benchmarks. The *y*-axis represents the normalized read and write latency. From this figure, we can first observe that FPC+SlidW slightly reduces read/write latency of most benchmarks. On average, compared with FPC, FPC+FNW and FPC+Space, SlidW decreases the write latency by 6.52%, 33.33% and 6.74%, respectively, and decreases the read latency by 2.78%, 3.60% and 2.80%, respectively. This shows that SlidW can induce a slight decrease in read/write latency of FPC, and can reduce the latency of the other existing works.

Then, we can observe that the write latency may be increased in some benchmarks when using the SlidW method. This is because write latency depends on the number of “0”s and “1”s to write, which are different in benchmarks. As the latency of writing “1” is larger than writing “0”, the more “1”s needed to write, the more write latency is needed. Furthermore, the more bits that need to be written, the more latency is needed. On the other hand, the read latency mainly depends on the change in the queue waiting time. When write latency changes, the queue waiting time changes, and the read time will also change.

In summary, these results show that SlidW does not induce obvious latency overhead compared with existing methods.

#### 5.2.7. Results of Energy Consumption

**Figure 16:**
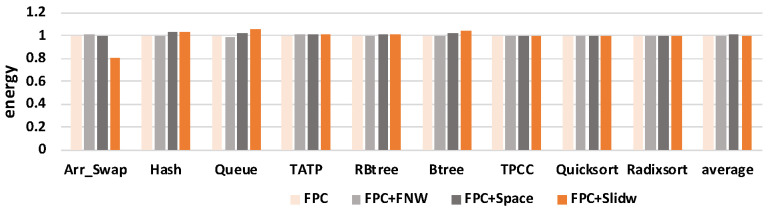
Comparison of energy for four different methods. Results are normalized to FPC.

This section presents the effect of SlidW on total energy involved in NVM. Figure 16 shows the results of energy with four methods in nine benchmarks. The *y*-axis represents the normalized energy.

As shown in this figure, compared with FPC and FPC+FNW, SlidW decreases the energy by 0.64% and 0.72% on average, respectively, as the energy is closely related to the number of bit flips. The calculation of energy includes two parts, one part is the energy required for preparation work when reading and writing data, and the other part is the energy required to flip the value on the cell; it needs to compare all the bits of the old and new data. If the value in the cell needs to change from “0” to “1”, the energy required for the set should be added, if the value changes from “1” to “0”, the energy of the reset should be added; if it does not change, do not add energy. These results also show that SlidW may induce more bit flips than FPC and FPC+FNW. However, the energy increase also happens in FPC+Space. Compared with the FPC+Space method, SlidW decreases the energy by 1.65% on average. For some benchmarks, such as *Queue* and *Btree*, SlidW consumes more energy than the other three methods. This may be because SlidW induces more bit flips by the frequent address changes. However, compared with the benefit of *local bit flip* reduction, this overhead on energy increase would be acceptable. In summary, the above results show that SlidW can achieve a 64.03% improvement on NVM lifetime and has little effect on energy consumption compared with the original FPC method.

## 6. Related Works

Existing works that study to improve the NVM lifetime can be categorized into two types. The first type is to reduce bit flips. FNW [15] reduced the bit flips by flipping whole word; the bit flip number between old and new words is more than half of the word. FlipMin [32] mapped each word to a set of vectors generated by the closest code and selected the vector that resulted in the smallest bit flip to encode the data. Alsuwaiyan et al. [33] improved FNW according to the characteristics of MLC/TLC NVM, making it more suitable for MLC/TLC NVM. Dan et al. [16] selectively used FNW and FlipMin based on the size left after compression. Kargar et al. [34] used a Hamming tree to map memory locations, directing writing similar-valued memory to reduce bit flips. Kargar et al. [35] used clustering on the written values based on similarity and then assigned the best-written memory location based on the value. In the method proposed by Chen et al. [14], the bit flips were reduced by replacing the newly written value with a floating-point number similar to the old value. Ho et al. [36] proposed an approximate write-once memory (WOM) code to reduce the number of writes to NVM. García et al. [12] proposed a replacement strategy to reduce last-level cache writes to memory and use compression to reduce writes.

There are also existing works that reduce bit flips for specific applications. Bittman et al. [37] made improvements to the hash list length, key-value and mapping method, and XOR the link list to reduce bit flips. Bittman et al. [13] used the XOR to turn the same part into 0, thus reducing bit flips. Staudigl et al. [38] reduced bit flips by writing data to adjacent memory cells. Ni et al. [11] used Shadow Paging to reduce unnecessary writes involved in logging. SlidW is orthogonal with these methods and can be used together with these to extend NVM lifetime.

The other method to extend NVM lifetime is to use wear leveling techniques to make memory blocks evenly worn. Most wear leveling-based works consider inter-block wear leveling. Huang et al. [8] detected the number of writes to each physical block and wrote data to the physical block with the fewest writes. Hakert et al. [9] used a red-black tree to estimate the age of blocks for wear leveling across pages. Xiao et al. [10] used the wear counter to dynamically adjust the use of NVM slots to achieve wear leveling between blocks. Qureshi et al. [39] examined wear leveling by moving written data from its original location to an adjacent location. Hakert et al. [40] studied wear leveling for B+-tree applications, and dynamically chose to store the data in DRAM or NVM. Kulandai et al. [41] used balanced gray codes to distribute the changes of a dirty bit across the whole Byte. As our SlidW method considers intra-block wear leveling, the work above can also work together with SlidW. In addition to the SlidW, some studies also perform intra-block wear leveling. Dgien et al. [42] conditionally wrote compressed data to the opposite end of the memory block to reduce wear. This method is similar to SlidW with a granularity of 2. Liu et al. [7] considered the intra-block wear leveling with a similar block division method with SlidW. However, its method does not fully consider the complicated situations on the left space and sizes of new and old data. We have already compared with SlidW in our experiment.

## 7. Conclusions

Compression algorithms have been widely used in NVM to further extend its storage capacity. This paper investigated its effect and observed that compression would increase or decrease bit flips. As the data size is smaller, the bit flips would only happen in a fixed range of area in the memory block, which would induce local wear effects. In order to better describe this effect, this paper proposes a new metric named *local bit flips*. Preliminary study results show that compression would cause increased *local bit flips*, i.e., local wear, which sacrifices the lifetime of NVM. This paper further proposes an intra-block wear leveling method to distribute the local wear effect across the whole block. Comprehensive experimental results show that SlidW can effectively reduce the number of *local bit flips* and improve NVM lifetime, with little overhead on energy consumption. Our work, SlidW, only considers the local wear effect induced by compression. In future work, we would combine the other lifetime extension methods, e.g., FNW, to study the combined wear effects of both compression algorithms and other lifetime extension methods.

## Figures and Tables

**Table 1:** Patterns in frequent pattern compression.

Word Patterns	Prefix	Compressed Size
zero run	000	0 bits
4-bit sign-extended	001	4 bits
1-byte sign-extended	010	8 bits
half-word sign-extended	011	16 bits
half-word padding with zero half-word	100	16 bits
two half-words with 1 Byte sign-extended in each	101	16 bits
repeated bytes	110	8 bits
uncompressed word	111	32 bits

**Table 2:** Patterns in BDI compression.

Patterns	Encode	Base Size (#*Byte*)	Delta	Compressed Size (#*Byte*)
Zeros	0000	1	0	1
repeated values	0001	8	0	8
Base8-Delta1	0010	8	1	16
Base8-Delta2	0011	8	2	24
Base8-Delta4	0100	8	4	40
Base4-Delta1	0101	4	1	20
Base4-Delta2	0110	4	2	36
Base2-Delta1	0111	2	1	34
Uncompressed	1111	N/A	N/A	64

**Table 3:** Tag calculation.

	*encode_tag*	*addr_tag*	*end_tag*
Case1	01	last written end_tag	addr_tag+ NSize
Case2	11	granularity-NSize	00
Case3	10	last written addr_tag	last written end_tag
Case4	11	granularity-NSize	00
Case5	00(uncompressed) or 01(compressed)	00	00

**Table 4:** Configuration of simulated system.

Processor and Cache	
CPU	single-core x86-64 processor, 1 GHZ
private L1/shared L2 caches	32 KB/2 MB
**Memory (PCM-Based Memory)**	
Capacity	8 GB, 1 channel, 1 rank, 8 banks
memory controller	first-ready-first-come-first-serve (FRFCFS)
set/reset lat.	60 cycles/20 cycles
read latency	54 cycles
**Parameters of SlidW**	
FNW en/decoding lat.	4 cycles/2 cycles
FPC compression/decompression lat.	8 cycles/5 cycles
Threshold $T_{size}$	48 Bytes
Threshold $T_{counter}$	3

**Table 5:** Benchmark information.

Benchmark	Description	Ops (#)	Writes
Array Swap	Swap items in an array	1,040,691	76.4%
Hash Table	Insert values to a hash table	2,870,832	18.6%
Queue	En/dequeue item to/from a queue	1,596,168	64.8%
TATP	Update records in TATP benchmark	6,360,544	54.4%
RBtree	Insert and delete nodes to a red-black tree	1,280,056	35.2%
Btree	Insert and delete nodes to a b-tree	4,378,578	33.6%
TPCC	Add new orders to the benchmark	1,532,425	50.2%
Quicksort	Sort numbers using key value	901,539	48.1%
Radixsort	Sort numbers using the DAC algorithm	1,046,992	44.3%

**Table 6:** Results of address change times and coverage size.

Benchmarks	Address Change Times		Coverage Size	
	FPC+Space	FPC+SlidW	FPC+Space	FPC+SlidW
Array Swap	143,275	505,578	3591	1283
Queue	233,886	608,865	3,735,788	932,333
TATP	50,566	375,543	1,103,508	601,245
Hash Table	171,335	167,383	787,772	369,626
Btree	400,678	822,446	899,457	65,736
RBtree	38,082	37,746	187,636	648
TPCC	2910	17,510	57,491	30,986
Quicksort	182	179	1431	565
Radixsort	183	195	1637	894

## Data Availability

No new data were created or analyzed in this study. Data sharing is not applicable to this article.
